# Alpha-Methylacyl-CoA Racemase (AMACR), a Potential New Biomarker for Glioblastoma

**DOI:** 10.3389/fonc.2020.550673

**Published:** 2020-10-13

**Authors:** Hyunji Lee, Minhee Kim, Seon-Hwan Kim, Quangdon Tran, Gyeyeong Kong, Chaeyeong Kim, So Hee Kwon, Jisoo Park, Jin Bong Park, Sungjin Park, Jongsun Park

**Affiliations:** ^1^Department of Pharmacology, Metabolic Syndrome and Cell Signaling Laboratory, Institute for Cancer Research, College of Medicine, Chungnam National University, Daejeon, South Korea; ^2^Department of Medical Science, College of Medicine, Chungnam National University, Daejeon, South Korea; ^3^Department of Neurosurgery, Institute for Cancer Research, College of Medicine, Chungnam National University, Daejeon, South Korea; ^4^College of Pharmacy, Yonsei Institute of Pharmaceutical Sciences, Yonsei University, Incheon, South Korea; ^5^Department of Life Science, Hyehwa Liberal Arts College, LINC Plus Project Group, Daejeon University, Daejeon, South Korea; ^6^Department of Physiology, College of Medicine, Chungnam National University, Daejeon, South Korea

**Keywords:** alpha-methylacyl-CoA racemase, glioblastoma, brain, cancer, biomarker

## Abstract

Alpha-Methylacyl-CoA racemase (AMACR), which was initially discovered as a prostate cancer marker, is critical for the chiral inversion mechanism of branched-chain fatty acids. However, the function of AMACR in brain tumors has not been investigated. In this study, AMACR appeared to be involved in glioblastoma. The protein and mRNA levels of AMACR were highly elevated in glioblastoma. Downregulation of AMACR inhibited cell proliferation. Comprehensive analysis of the public REMBRANDT GBM dataset also confirmed that the level of AMACR expression was correlated with the clinical prognosis of glioma patients. In summary, these findings indicate that AMACR expression is increased in a glioblastoma cell line and glioma patients, suggesting that AMACR might be a potential diagnostic marker and therapeutic target for cancer, including glioma.

## Introduction

Glioblastoma multiform (GBM) is associated with poor prognosis and frequent relapse (1). Almost all glial neoplasms are astrocytic tumors. Approximately 80% of astrocytic tumors belong to glioblastomas with common genetic mutations (2). Treatment strategies for gliomas are open surgery, radiotherapy, and chemotherapy. However, these treatments do not guarantee a survival rate of more than 15 months (3). Glioma stem cells are resistant to radiotherapy and chemotherapy, which increases tumor aggressiveness and the chance of treatment failure. Therefore, it is necessary to identify biomarkers that can diagnose GBM. A well-known marker for glioma stem cells is CD133; however, glioma stem cells are also found in CD133-negative cells. To overcome this limitation, additional biomarkers of glioma stem cells are required. To date, biomarkers such as CD44, Integrin-α6, CD15, L1 cell adhesion molecule, CD90, and A2B5 have been found in glioma stem cells (1).

Alpha-Methylacyl-CoA racemase (AMACR) is a marker of carcinoma stem cells. AMACR has emerged as a prostate cancer marker, and its expression is high in other cancers such as colon cancer, liver cancer, and renal cancer, and it is a potential target of anti-cancer drugs (4). Several AMACR inhibitors have been developed as potential cancer treatments. AMACR is a peroxisomal and mitochondrial enzyme that plays a key role in the metabolism of cholesterol and branched-chain fatty acids. The function of AMACR in cancer is associated with lipid metabolism and the activity of nuclear receptors such as farnesoid X receptor and peroxisome proliferation activation receptor and expression of cyclooxygenase-2 (5). In this study, we investigated the potential of AMACR as a biomarker in brain tumors. The correlation between human brain cell lines and brain tumor tissues was analyzed using histological observation and cell biological levels.

## Materials and Methods

### Antibodies and siRNAs

The following antibodies were used: P504S from Santa Cruz Biotechnology (Santa Cruz, CA, USA); AMACR from Thermofisher scientific (Pittsburgh, PA, USA); β-actin from Sigma-Aldrich (St. Louis, USA). Horseradish peroxidase-conjugated anti-mouse IgG or anti-rabbit IgG secondary antibodies were purchased from Komabiotech (Seoul, Korea). The siRNA against human AMACR were synthesized by Cosmogenetech co, Ltd (Seoul, Korea). The siRNA sequences for AMACR were the following: sense 5′- GAGAUUUAUCAGCUUAACUTT-3′ and anti-sense 5′-AGUUAAGCUGAUAAAUCU-CTT-3′. The siRNA sequences for control were the following: sense 5′-UUCUCCGA-ACGUGUCACGUTT-3′ and anti-sense 5′-ACGUGACACGUUCGGAGAATT-3′.

### Cell Culture and Transfection

The glioblastoma cells (U87-MG, U251-MG, and U343-MG) were maintained in medium (RPMI) supplemented with 10% (v/v) FBS, 25 mM HEPES (Thermo Fisher Scientific), 1% (v/v) Antibiotics-Antimycotics (Life Technologies). U87-MG cells and U251-MG cells were transiently transfected with 30 nM control siRNA or AMACR siRNA by using Neon Transfection System (Invitrogen, Carlsbad, CA, USA).

### Patient Samples

The experiment for immunohistochemistry was approved by the Hospital Institutional Review Board (approval number CNUH 2018-03-014) according to the Declaration of Helsinki at Chungnam National University Hospital (Daejeon, Korea). The biospecimens and data used for this study were provide by the Biobank of Chungnam National University Hospital, a member of the Korea Biobank Network.

### Immunoblot Analysis

The immunoblot analysis was performed as the described previously (6, 7). Briefly, cells were placed on ice and extracted with lysis buffer containing 50 mM Tris-HCl, pH 7.5, 1% (v/v) Nonidet P-40, 120 mM NaCl, 25 mM sodium fluoride, 40 mM β-glycerol phosphate, 0.1 mM sodium orthovanadate, 1 mM phenylmethylsulfonyl fluoride, 1 mM benzamidine-HCl, and 2 μM microcystin-LR. Lysates were centrifuged for 15 min at 12,000 g. The cell extracts were resolved by 10–15 % (v/v) SDS-PAGE, and transferred to Immobilon-P membranes (Millipore). The filters were blocked for 1 h in 1 X tri-buffered saline buffer (TBS; 140 mM NaCl, 2.7 mM KCl, 250 mM Tris- HCl, pH 7.4), containing 5% (w/v) skimmed milk and 0.2% (v/v) Tween-20, followed by an overnight incubation with the anti-AMACR and anti-Actin antibodies diluted 1,000-fold at 4°C. The secondary antibody was horseradish peroxidase-conjugated anti-mouse IgG or anti-rabbit IgG (Komabiotech), diluted 5,000-fold in the blocking buffer. Visualization was achieved with chemiluminescence through X-ray film exposure (Agfa-Gevaert N.V, Morstel, Antwerp, Belgium).

### Real-Time Quantitative Reverse Transcription-Polymerase Chain Reaction (qRT-PCR)

Total RNA was extracted from frozen tissue samples or from cells using the PureHelix RNA Extraction Solution (Nanohelix, South Korea). The cDNA was synthesized from total RNA with the SuperScript III First-Strand Synthesis System for qPCR (Invitrogen, Grand Island, USA). The qPCR measurement of individual cDNAs was performed using SYBR green dye to measure duplex DNA formation with the StepOne Plus real-time PCR system (Invitrogen) and normalized to the expression of glyceraldehyde 3-phosphate dehydrogenase (GAPDH) RNA. The following primers were used in the qPCR (F: Forward, R: Reverse); human AMACR: F-5′-GCTGGCCACGATATCAACTAT / R−5′-GCTTCCCACAGACTCGATTT; human GAPDH: F-5′-TCGACAGTCAGCCGCATCTTCTTT / R-5′-TACGACCAAATCCGTTG ACTCCGA.

### RNA Sequencing and RNA-Seq Data Analysis

Total RNA of U87-MG, U251-MG and normal brain was extracted using Trizol reagent (Invitrogen) following the manufacturer's procedures. The total RNA quantity and purity were analysis of Bioanalyzer 2100 and RNA 6000 Nano LabChip Kit (Agilent, Santa Clara, USA). Roughly 10 μg of total RNA was subjected to isolate Poly (A) mRNA with poly-T oligo attached magnetic beads (Invitrogen). Following purification, the mRNA is fragmented into small pieces using divalent cations under raised temperature. Then the cleaved RNA fragments were reverse-transcribed to create the final cDNA library in accordance with the protocol for the mRNA-Seq sample preparation kit (Illumina). The average insert size for the paired-end libraries was 300 bp (± 50 bp). Next we performed the paired-end sequencing on an Illumina Hiseq 2,000 system at Macrogen (Seoul, Korea) following the vendor's recommended protocol. For each sample, sequenced reads were aligned to the UCSC human reference genome (http://genome.ucsc.edu/) using the Tophat package (http://ccb.jhu.edu/software/tophat), which initially removes a portion of the reads based on quality information accompanying each read and then maps the reads to the reference genome. FPKM (fragments per kilo base of exon per million fragments mapped) were calculated to compare the expression level of AMACR mRNA variants in each sample.

### Immunofluorescence

U343-MG cells were grown on glass coverslips until they were 50–70% confluent. After 24 h, the cells were fixed in 4% (w/v) paraformaldehyde at room temperature for 10 min and permeabilized in 0.2% (v/v) Triton X100 for 5 min at room temperature. Then cells were incubated in blocking buffer containing 5% (v/v) bovine serum albumin (Sigma) in 1 X TBS for 1 h at 37°C. The rabbit polyclonal anti-AMACR was diluted 200-fold for primary antibody and incubated for overnight. The secondary antibody, FITC-conjugated anti-rabbit antibody (BD Biosciences) was used. After appropriate rinsing, cover slips were mounted with Vectashield (Vector Laboratories) and visualized using a Leica confocal microscope.

### Immunohistochemistry

The analysis of immunohistochemistry was performed as the described previously (8). A human cancer tissue array slide with paraffin sections was purchased from Bio Max (US Biomax Inc). Histostain-Plus kits (Zymed Laboratories Inc.) were used in accordance with the manufacturer's instructions for the immunohistochemistry of tissue array. Briefly, paraffin sections were deparaffinized with xylene and rehydrated in a graded series of ethanol. The slide was submerged in peroxidase quenching solution for 10 min. After it was washed twice with PBS for 5 min, it was added with 2 drops of Reagent A for blocking and incubated for 30 min. Following two washes with PBS, the primary antibody was applied at 4°C for overnight. Then biotinylated secondary antibody, Reagent B, was added after rinsing with PBS. It was incubated at room temperature for 1 h. It was rinsed with PBS and dropped with enzyme conjugated Reagent C. After it was washed with PBS, DAB chromogen, and a mixture of Reagent D1, D2, and D3, it was dropped, and signals were observed with a fluorescence microscope. Then the reaction was stopped with distilled water, and pictures were taken with a microscope.

### Bioinformatics Data Set

Glioma data sets and corresponding clinical data were downloaded from the publicly available databases (524 cases from the REMBRANDT cohort GBM dataset; http://www.betastasis.com/glioma/tcga_gbm/).

### Real-Time Assay for Cell Proliferation

Real-time assay for cell proliferation was measured using an xCELLigence RTCA DP system (Roche Applied Science, Indianapolis, IN, USA), which monitors cellular events in real-time without the incorporation of labels. Briefly, cells were placed into well of an E-plate 16 (U343-MG; 3x10^3^ cells) and incubated for indicated times.

### Statistical Analysis

Data are expressed as the mean ± S.D. from at least three separate experiments performed in triplicate. The differences between groups were analyzed using a Student's *t* test and *p* < 0.05 (^*^) was considered significant, and *p* < 0.01 (^**^) was highly significant compared with corresponding control values. Statistical analyses were carried out using SPSS software ver. 13.0 (SPSS Inc.).

## Results

### AMACR Expression Is High in Glioblastoma Tissue Samples

**Figure 1:**
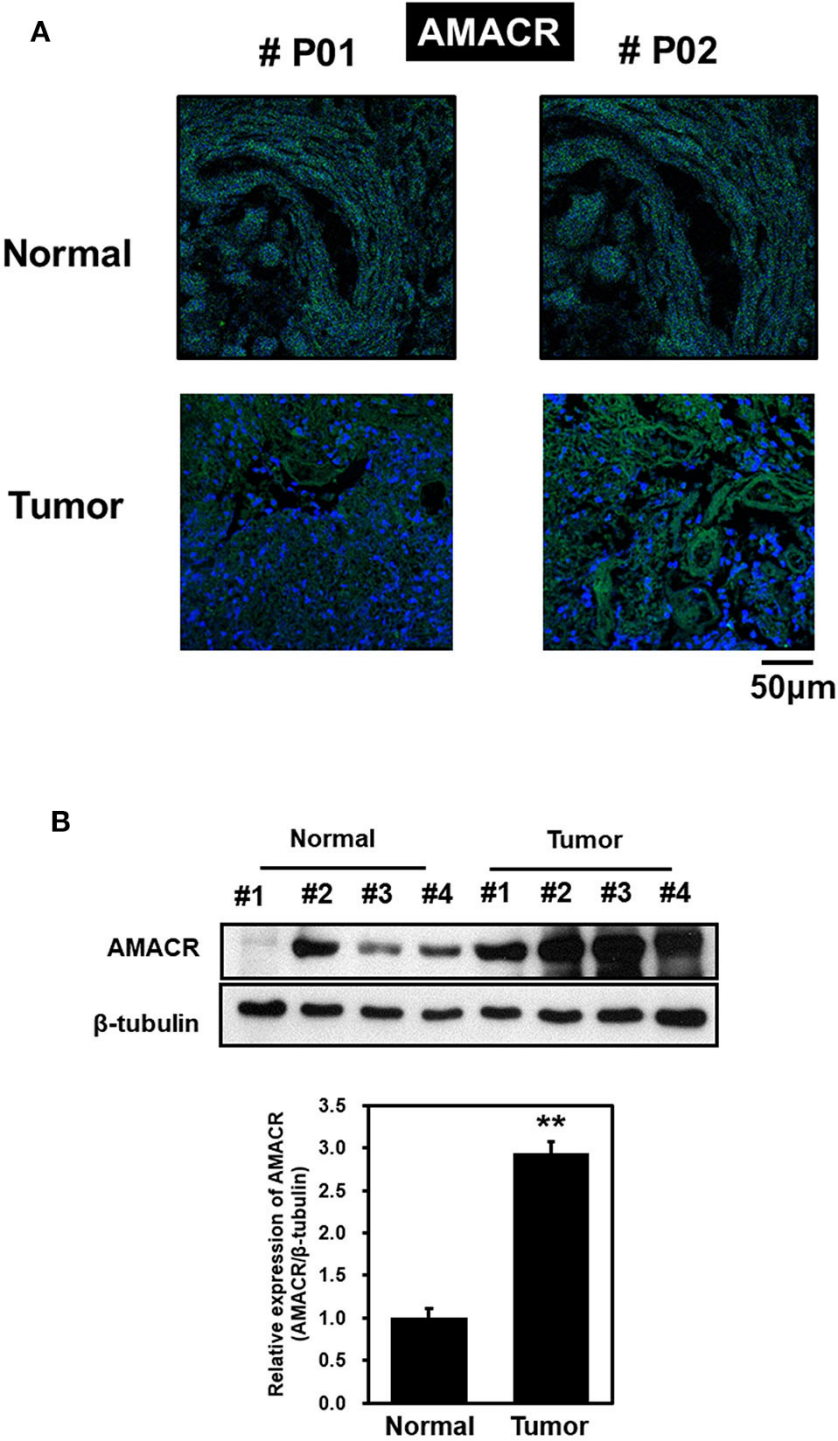
AMACR expression levels in human brain tumors **(A)** Human glioma tissue arrays were analyzed by immunofluorescence (IF) for AMACR staining. Representative images from two patients (#P01 and #P02) were shown. Green, AMACR; blue, DAPI;, Scale bars, 50 μm. **(B)** Total cell lysates from normal and GBM tissues (no. 1–4) from patient were analyzed for AMACR expression (top panel). Tumor-associated normal tissue (Normal) served as a control. Relative densities were calculated by densitometry. Relative differences in AMACR expression levels were determined by normalizing all densitometric values to those of beta-tubulin (in each lane) and setting the control values to 1 (bottom panel). The results are presented as means ± SD of data from two independent experiments. *p < 0.05, **p < 0.01.

To explore the possible involvement of AMACR in clinical samples of brain cancer, immunofluorescence (IF) analysis with tissue array was performed. IF analysis with an AMACR antibody in tumor tissue showed an increase in AMACR levels compared to normal tissues (Figure 1A). In addition, total cell lysates from normal and cancerous tissues derived from GBM patients during surgery were analyzed by immunoblotting using an AMACR antibody. As expected, expression of AMACR in GBM tissues was drastically increased compared to normal tissues, suggesting that AMACR expression is upregulated in glioblastoma patients (Figure 1B).

### Enhancement of AMACR Expression in Glioblastoma Cell Lines

**Figure 2:**
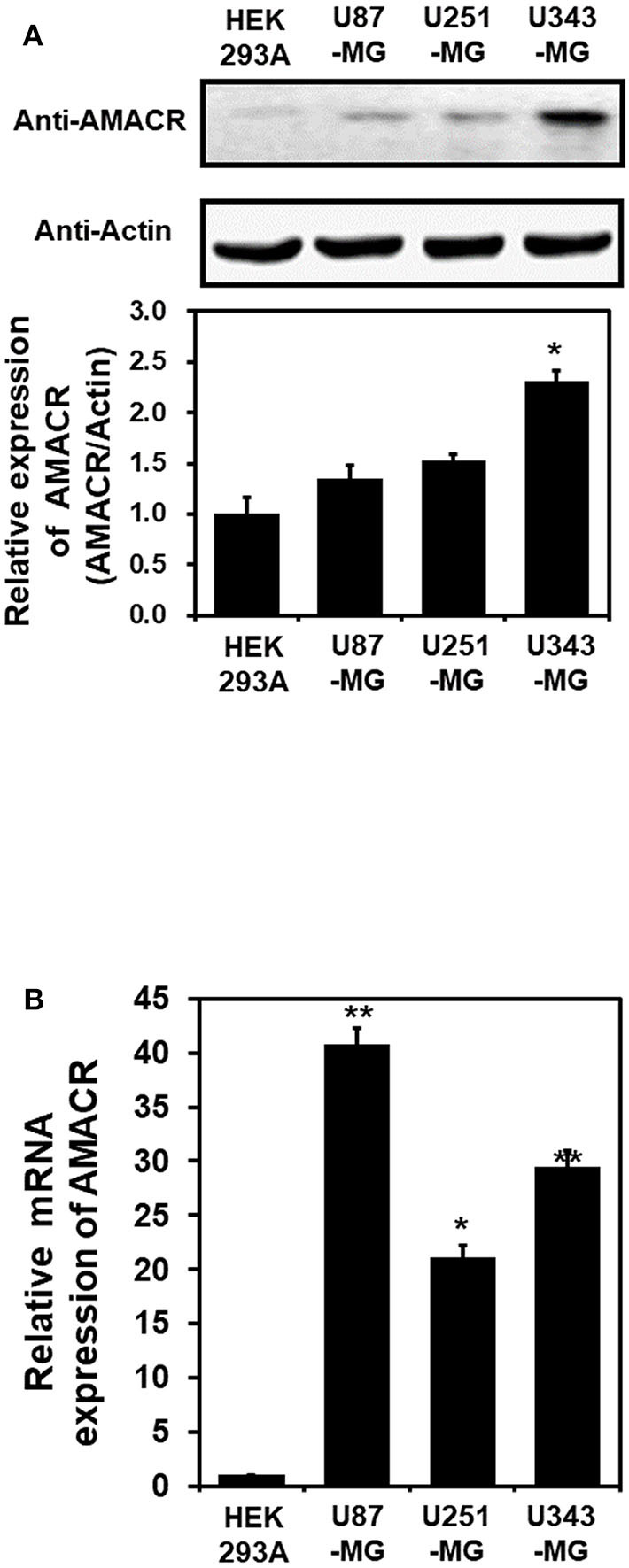
AMACR expression in glioblastoma cell lines **(A)**. For immunoblot analysis with AMACR and actin antibodies, cell lysates were isolated from 3 established GBM cell lines (U87-MG, U251-MG, and U343-MG) and one established non-GBM cell lines (HEK-293A). The results are representative of 3-independent experiments (top panel). Relative density was obtained by densitometry of the corresponding immunoblot data. Relative and statistical differences of AMACR expression were determined by normalizing values for actin in each lane and set the values for HEK-293A as 1 (bottom panel). The results are presented as means ± S.D. of three independent experiments. **(B)** Extracted total RNA from each GBM cell line was analyzed using human AMACR specific primer sets by real-time quantitative polymerase chain reaction (qPCR), as described in the Materials and Methods section. The results are presented as means ± S.D. of three independent experiments. *p < 0.05, **p < 0.01.

To investigate the putative roles of AMACR in brain cancer, immunoblot analysis with the AMACR antibody was performed. The protein expression of AMACR was significantly increased in U343-MG cells compared to HEK293A cells (Figure 2A). Quantitative real-time PCR analysis with glioblastoma cell lines also showed that the AMACR mRNA level was increased in U87-MG, U251-MG, and U343-MGcells compared to HEK293A cells (Figure 2B).

### Transcriptional Induction of AMACR mRNA in U87-MG Cells and U251-MG Cells

**Figure 3:**
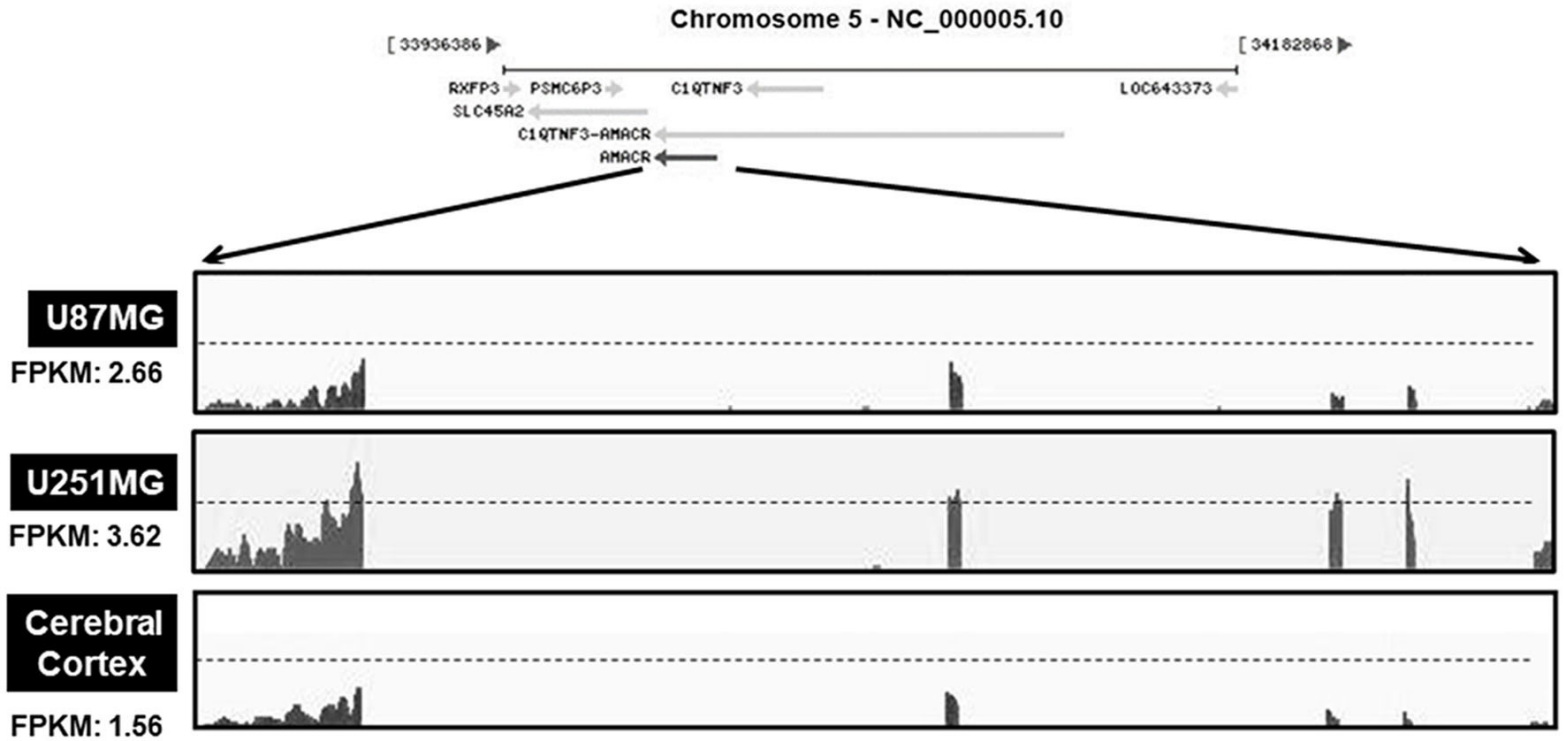
Relative changes of AMACR transcripts from GBM cells in standard RNA-seq data. Total RNA were isolated from two GBM cell lines (U87-MG and U251-MG) and normal brain tissue. These samples were further analyzed by the standard RNA deep sequencing (RNA-seq) as described in the material and methods. RNA-seq read density for AMACR transcripts was plotted with the relative RNA-seq read coverage (counts). Fragments per kilobase of exon per million fragments mapped (FPKM) were calculated to compare the expression level of AMACR mRNA variants in each sample.

Based on the results presented in Figures 1, 2, the mRNA levels of AMACR were examined using transcriptome profiling with glioblastoma cell lines. Total RNA was isolated from two cell lines (U251-MG and U87-MG). The isolated mRNA from total RNA was split into small fragments to make the final cDNA library. Fragments per kilobase of exon, per million fragments mapped (FPKM) were considered as AMACR mRNA levels in each sample. FPKM values were noticeably higher in U87-MG cells (2.66) and U251-MG cells (3.62) than in the cerebral cortex (1.56) (Figure 3), demonstrating that AMACR transcription is enhanced in glioblastoma cells.

### Cytoplasmic Localization of AMACR and Effects of AMACR on Cell Proliferation in U343-MG Cells

**Figure 4:**
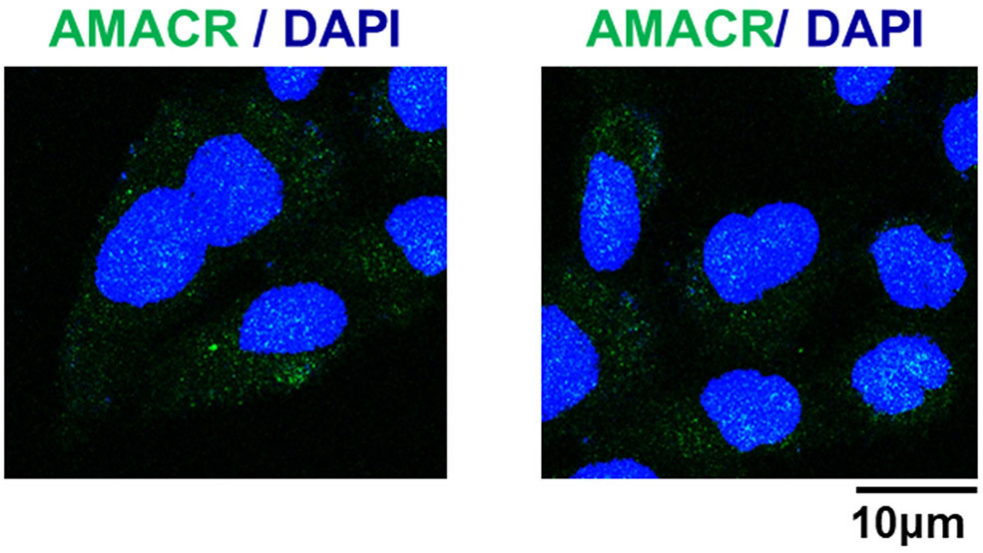
Subcellular localization of AMACR in U343-MG cells. U343-MG cells were grown on glass coverslips, fixed, and permeabilized with 0.2% (v/v) Triton X-100. After immunostaining with anti-AMACR antibody, the cover slips were mounted on Vectashield and examined using a Zeiss confocal microscope. Scale bars: 10 μm.

To further evaluate the role of AMACR, the intracellular localization of AMACR was examined in U343-MG cells by IF imaging. Interestingly, AMACR was located in both the nucleus and cytosol in a dot-like pattern (Figure 4), indicating that AMACR showed ubiquitous localization in glioblastoma cells.

### Downregulation of Cell Proliferation by AMACR-Knockdown in U343-MG Cells

**Figure 5:**
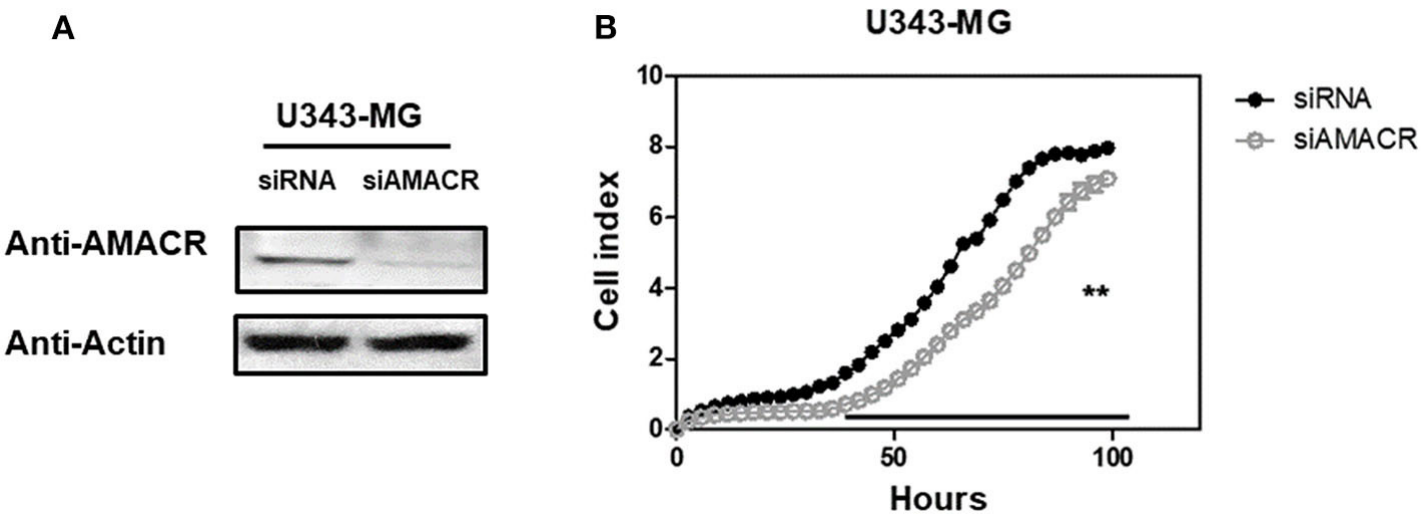
Effects of AMACR on cell proliferation in GBM U343-MG cells. **(A)** U343-MG cells were transiently transfected with negative control-siRNA (siNC) or AMACR-siRNA (siAMACR). After 72 h-transfection, cell lysates were analyzed by western blotting with AMACR antibody and β-actin antibody. **(B)** The proliferation of knock downed cells were measured using an xCELLigence system in U343-MG cells (right panel) for 100 h. Values are the means ± SEM (*n* = 4).

To further characterize the AMACR function in U343-MG cells, siRNA-mediated knockdown and real-time cell analysis were employed. AMACR was depleted after the treatment of cells with siAMACR (Figure 5A). An *in vivo* cell proliferation assay revealed that siRNA-mediated knockdown of AMACR in U343-MG cells inhibited cell proliferation (Figure 5B). Taken together, these findings suggest that AMACR has an important function in cancer cells.

### AMACR Expression Is Associated With Poor Prognosis in Glioma of a REMBRANDT Cohort

**Figure 6:**
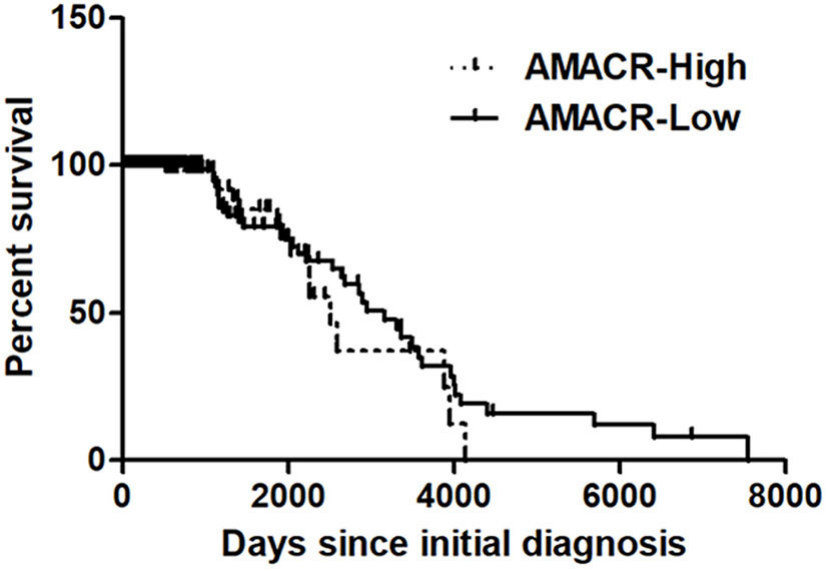
Kaplan–Meier's analysis for overall survival based on AMACR expression in glioma specimens of the REMBRANDT cohort. The Kaplan-Meier curve compares the level of AMACR mRNA expression with overall survival. *P* values were obtained using the log-rank test, and hazard ratios (HRs) with 95% confidence intervals (CIs) were determined using the aid of a univariate Cox's regression model.

To further evaluate the previous findings (Figures 1–5), overall survival curves were calculated using the Kaplan–Meier method and compared using a log-rank test with glioma of the REMBRANDT cohort (http://www.betastasis.com/glioma/Rembrandt/). High AMACR mRNA expression (in 208 patients), compared with low expression (in 121 patients), was significantly associated with poor survival (log-rank *P* value = 0.015; HR = 1.30, 95% confidence interval = 0.68–2.49) (Figure 6), indicating that the AMACR expression level is correlated with the clinical prognosis of glioma patients.

## Discussion

Our study provides evidence that AMACR, a known prostate cancer marker, might also be a prognostic marker for GBM. We identified the presence of AMACR in GBM. Protein levels of AMACR were increased in glioma patients (Figure 1), and mRNA and protein levels of AMACR were increased in glioma cell lines (Figures 2, 3). Interestingly AMACR was localized with a dot-like pattern in both the nucleus and cytosol (Figure 4). Downregulation of AMACR inhibited cancer cell growth (Figure 5). Bioinformatic analysis showed that high mRNA levels of AMACR were significantly associated with poor survival in glioblastoma (Figure 6). AMACR promotes cancer progression through fatty acid β-oxidation (9, 10). Thus, AMACR may be an important target for anti-cancer therapeutics.

Several AMACR inhibitors have been developed, such as 2-methylacyl-CoA analogs containing fluorine on the β-carbon 2 or in the methyl group 1, including 2-(trifluoromethyl)-tetradecanoyl-CoA, (3R, 2S)-3-fluoro-2-methylhexadecanoyl-CoA, E-13-iodo-2-methylenetridec-12-enoyl-CoA, ebselen, ebselen oxide, and 2-(2,5-dihydroxy-4-methylphenyl)-5-methylbenzene-1,4-diol (5). AMACR has also been studied as a target for peptide vaccines. AMACR has four HLA-A24 (human leukocyte antigen A24)-binding motifs. Peptides derived from these motifs (NYLALSGVL, NMVEGTAYL, FYELLIKGL, and IYQLNSDKII) increased the production of cytotoxic T lymphocytes that kill cancer cells in prostate cancer patients with high AMACR levels (11).

Gliomas, in particular GBM, are the most aggressive cancers, which means these tumors can grow fast and spread quickly. The five-year survival rate for GBM patients is only 4–5% and the average survival time is 12.6 months (12). New approaches to increase the overall survival of GBM patients, such as monoclonal antibodies, cancer vaccines, checkpoint inhibitors, oncolytic viruses, and adoptive cells, are undergoing clinical trials (13). The short survival time of GBM patients can be attributed to treatment limitations, and multiple biomarkers need to be explored for patient-specific treatments. Our findings support AMACR as a potential biomarker in GBM patients and a potentially important target for anti-cancer drugs.

## Data Availability Statement

The datasets presented in this study can be found in online repositories. The names of the repository/repositories and accession number(s) can be found below: https://www.ncbi.nlm.nih.gov/sra/PRJNA657687.

## Ethics Statement

The experiment for immunohistochemistry was approved by the Hospital Institutional Review Board (approval number CNUH 2018-03-014) according to the Declaration of Helsinki at Chungnam National University Hospital (Daejeon, Korea). The biospecimens and data used for this study were provide by the Biobank of Chungnam National University Hospital, a member of the Korea Biobank Network. The patients/participants provided their written informed consent to participate in this study.

## Author Contributions

HL, MK, S-HK, SP, and JoP: contributes to conception and design. HL, MK, CK, QT, JBP, JoP, and JiP: acquisition of data, or analysis and interpretation of data. HL, MK, GK, JBP, SHK, and SP: contributions to assist the exam and acquisition of data. All authors agreed to be accountable for all aspects of the work and all authors read and approved the final manuscript.

## Conflict of Interest

The authors declare that the research was conducted in the absence of any commercial or financial relationships that could be construed as a potential conflict of interest.

## References

[1] Glaser T Han I Wu L Zeng X. Targeted nanotechnology in glioblastoma multiforme. Front Pharmacol. (2017) 8:166. 10.3389/fphar.2017.00166 28408882 PMC5374154

[2] Han L Zhang A-L Xu P Yue X Yang Y Wang G-X. Combination gene therapy with PTEN and EGFR siRNA suppresses U251 malignant glioma cell growth *in vitro* and *in vivo*. Med Oncol. (2010) 27:843–52. 10.1007/s12032-009-9295-8 19728186

[3] Virk SM Gibson RM Quinones-Mateu ME Barnholtz-Sloan JS. Identification of variants in primary and recurrent glioblastoma using a cancer-specific gene panel and whole exome sequencing. PLoS One. (2015) 10:e0124178. 10.1371/journal.pone.0124178 25950952 PMC4423782

[4] Shukla N Kumar Adhya A Rath J. Expression of alpha - methylacyl - coenzyme a racemase (AMACR) in colorectal neoplasia. J Clin Diagn Res. (2017) 11:EC35–8. 10.7860/JCDR/2017/25303.9727 28571147 PMC5449793

[5] Na A-Y Yang E-J Jeon JM Ki SH Song K-S Lee S. Protective effect of isoliquiritigenin against ethanol-induced hepatic steatosis by regulating the SIRT1-AMPK pathway. Toxicol Res. (2018) 34:23–9. 10.5487/TR.2018.34.1.023 29371998 PMC5776912

[6] Lloyd MD Yevglevskis M Ling Lee G Wood PJ Threadgill MD Woodman TJ. α-Methylacyl-CoA racemase (AMACR): metabolic enzyme, drug metabolizer and cancer marker P504S. Prog Lipid Res. (2013) 52:220–30. 10.1016/j.plipres.2013.01.001 23376124

[7] Tran Q Jung J-H Park J Lee H Hong Y Cho H. S6 kinase 1 plays a key role in mitochondrial morphology and cellular energy flow. Cell Signal. (2018) 48:13–24. 10.1016/j.cellsig.2018.04.002 29673648

[8] Na CH Hong JH Kim WS Shanta SR Bang JY Park D. Identification of protein markers specific for papillary renal cell carcinoma using imaging mass spectrometry. Mol Cells. (2015) 38:624–9. 10.14348/molcells.2015.0013 26062552 PMC4507028

[9] Batash R Asna N Schaffer P Francis N Schaffer M. Glioblastoma multiforme, diagnosis and treatment; recent literature review. Curr Med Chem. (2017) 24:3002–9. 10.2174/0929867324666170516123206 28521700

[10] Kuzmanov A Hayrabedyan S Karaivanov M Todorova K. Basal cell subpopulation as putative human prostate carcinoma stem cells. Folia Histochem Cytobiol. (2007) 45:75–80. 17597019

[11] Ouyang B Leung Y-K Wang V Chung E Levin L Bracken B. α-Methylacyl-CoA racemase spliced variants and their expression in normal and malignant prostate tissues. Urology. (2011) 77:249.e1–7. 10.1016/j.urology.2010.08.005 21195844 PMC3051191

[12] Honma I Torigoe T Hirohashi Y Kitamura H Sato E Masumori N. Aberrant expression and potency as a cancer immunotherapy target of alpha-methylacyl-coenzyme A racemase in prostate cancer. J Transl Med. (2009) 7:103. 10.1186/1479-5876-7-103 20003233 PMC2797764

[13] Carlsson SK Brothers SP Wahlestedt C. Emerging treatment strategies for glioblastoma multiforme. EMBO Mol Med. (2014) 6:1359–70. 10.15252/emmm.201302627 25312641 PMC4237465

